# Dataset for performance of superior dairy cattle sires based on daughter's milk yield in tropical country

**DOI:** 10.1016/j.dib.2024.110161

**Published:** 2024-02-09

**Authors:** Prafangasti Sarah Ginantika, Didin Supriat Tasripin, Heni Indrijani, Dedi Ruswandi

**Affiliations:** a Animal Husbandry, Graduate School, Universitas Padjadjaran, Sumedang, Jatinangor 45363, Indonesia; b Departement of Livestock Production, Faculty of Animal Husbandry, Universitas Padjadjaran, Sumedang, Jatinangor 45363, Indonesia; c Departement of Agronomy, Faculty of Agriculture, Universitas Padjadjaran, Sumedang, Jatinangor 45363, Indonesia

**Keywords:** Breeding value, Dairy cattle, Heritability, Sires

## Abstract

Sire has an important role because they could have more offspring than dam does. The research aims to determine the production performance of Friesian Holstein, estimation of heritability values, estimation of breeding values, and ranking of dairy cattle sires. The objects of the study were the complete record of milk production, lactation length, lactation peak, and dry period length from first to fourth lactation from 2017-2021. This study used the descriptive method. The results of the research showed that productivity performance is great, 1st lactation milk production was 8,029.28±1,112 kg, the lactation length was 321.26±38.48 days, the condition of average peak production was obtained on day 85.35±29.25 with milk production of 32.55±4.16 kg, and dry period length was 51.37±9.33 days. 2nd Lactation milk production was 7,761.66±1,145, the lactation length was 323.66±43.06 days, the condition of average peak production was on day 58.43±21.11 with milk production of 40.79±5.30 kg and the dry period length was 65.10±22.69 days. 3rd lactation milk production 3 was 7,788.92±1,148 kg, the lactation length was 326.64±46.74 days, peak production was on day 61.88 ±22.72 with milk production of 43.62±5.11 kg; the dry period length was 65.00±20.49 days. 4th Lactation milk production was 7,484.18±1,133 kg, lactation length was 323.04±42.23 days, peak production was on day 66.39±24.26 with the milk production of 43.82±5.68, the dry period length was 65.78±21.60 days. The estimated heritability value for milk production, 0.03 ± 0.02, is included in the low category. The ranking of 10 sires that have the potential to increase the genetic based on their estimated breeding value is 595.91 kg (O.S.Elmer-XA), 264.16 kg (M.Z.Merlin.-XA), 252.38 kg (L.Muscadet-XA), 247 .12 kg (C.Toyjet), 239.01 kg (S. Gypsy B), 214.82 kg (WestCoastPldge), 188.14 kg (M.Z.Merlin-ET), 178.56 kg (Brasilia), 166.43 kg (L.JetBowser-XA), 162.06 kg (MRMUDD-XA).

Specifications Table

**Table:** 

Subject	Data Article (Animal Science)
Specific subject area	Animal Breeding, Male Breeding Soundness, Breeding value
Data format	**Analyzed data**
Type of data	Table and Figure
Data collection	The object of the study was the complete record of milk production, lactation length, peak production, and dry period length of FH cows in lactation 1 to 4. Data were collected by using all data contained in the company Ultra Farm South Bandung in 2017-2021 with the census method, then data were descriptively and quantitatively analysed.
Data source location	City/Town/Region: Bandung, West Java, Country: Indonesian
Data accessibility	Repository name: Mendeley Data Data identification number: 10.17632/2sm93h8t7y.2 Direct URL to data: https://data.mendeley.com/datasets/2sm93h8t7y/2

## 1 Value of the Data

• The data set in this article provides information on the productivity performance of dairy cattle in Indonesia, heritability values, and estimated breeding value of sire's
• Estimation of sire's breeding value can be used to select dairy cow parents based on sire herd rank
• The data set and do files will enable other researchers to replicate the current study and to carry out extended analyses of an estimated breeding value of dairy cattle sire's

## 2 Background

Dairy cows are one of the milk-producing livestock contributing to meeting national milk demand in Indonesia. Friesian Holstein (FH) cattle are adaptable, have high production, and are commonly kept in Indonesia [1]. The availability of dairy sires in Indonesia still relies on imports from Australia, New Zealand, and other countries producing superior dairy sires in the form of sire's stock and frozen semen. Through import activities, it is hoped that the superior characteristics of these sires can be passed on to their offspring. Sires cannot produce milk, so production capacity is estimated from the production of daughters' offspring based on production performance records. Productivity performance parameters are milk production, length of lactation, peak production, and dry period length. Increasing milk production must be accompanied by a selection program, namely choosing cows that will serve as dams or sires. The selection of prospective sires is much more important in increasing milk production than the selection of prospective replacement dams. This is because sires will have far more offspring than a dam, with the frozen semen technique in artificial insemination. The selection carried out is based on estimated genetic quality or breeding value by taking into account genetic parameters such as heritability, thereby producing calves that have a superior genetic quality that is higher than the average of their dams or sires. Each sire has a different breeding value, so estimating the breeding value of dairy cattle is important, considering the output of raising dairy cattle to produce superior breeds.

## 3 Data Description

This article examines the livestock productivity, heritability, and breeding values of bulls. This study's object includes animal identity data, pedigree data, and milk production performance records for Holstein Friesian dairy cows born between 2017-2021. This study's parameters included milk yield, length of lactation, peak production, and dry length period. Another factor to consider is the environmental state. High air temperatures have a negative impact on dairy cow welfare and production across Europe, especially tropical regions. This causes severe cattle losses [2,3]. Humidity, air velocity, and insolation all have a major impact on animal thermoregulation [4,5]. Special indices are employed to measure the overall impact of various environmental conditions on animals. The temperature-humidity index (THI) is commonly used to assess how ambient temperature and relative humidity affect animals [6]. Genetic advancement on selection indicators in dairy farming by 90–95% depends on the amount of breeding value of bull breeders. Realizing how breeding value (BV) is passed down across generations is crucial for selecting the right pairs and advancing the breed [7]. Accurate and detailed forecasts help farmers decrease economic risks when selecting genetic material [8]. Estimation of breeding value uses the BLUP method, which has been widely used in various countries. This method is the most accurate estimated breeding value of sire compared to other methods [9].

The results of the productivity performance of FH dairy cows in the first to fourth lactation are presented in the table. Real and corrected milk production is presented in Table 1, lactation length is presented in Table 2, peak production is presented in Table 3, and dry length period is presented in Table 4.

**Table 1:** Total milk yield and corrected milk yield of FH Cows in 1^st^, 2^nd^, 3^rd^, and 4^th^ lactation.

Description	Lactation 1	Lactation 2	Lactation 3	Lactation 4
Total Milk Yield				

Average (Kg)	7,770.55	8,607.05	9,292.79	9,157.50
Maximum (Kg)	13,358.00	14,694.00	15,591.61	14,064.07
Minimum (Kg)	4,692.00	4,714.66	5,270.92	5,622.46
Standard of Deviation	1,402,12	1,625.51	1,782.72	1,652.86
Coefficient of Variation (%)	18.04	18.89	19,18	18,05

Corrected Milk Yield	Lactation 1	Lactation 2	Lactation 3	Lactation 4

Average (Kg)	8,029.28	7,761.66	7,788.92	7,484.18
Maximum (Kg)	11,272.20	12,118.82	12,330.56	10,911.11
Minimum (Kg)	4,999.52	4,254.33	4,631.42	4,855.54
Standard of Deviation	1,112.95	1,145.82	1,148.99	1,133.25
Coefficient of Variation (%)	13,86	14,76	14,75	15,14

**Table 2:** Length of lactation of FH Cows in 1^st^, 2^nd^, 3^rd^, and 4^th^ lactation.

Length of lactation	Lactation 1	Lactation 2	Lactation 3	Lactation 4
Average (days)	321.26	323.66	326.64	323.04
Maximum (days)	432.00	436.00	452.00	431.00
Minimum (days)	255.00	215.00	228.00	249.00
Standard of Deviation	38.48	43.06	46.74	42.23
Coefficient of Variation (%)	11.98	13.30	14.31	13.07

**Table 3:** Peak production of FH Cows in 1^st^, 2^nd^, 3^rd^, and 4^th^ lactation.

	Peak Production (days)				Peak Production (Kg)				
Lactation	1	2	3	4	1	1	2	3	4
Average	85.35	58.43	61.88	66.39		32.55	40.79	43.62	43.82
Maximum	155.00	117.00	119.00	124.00		49.40	75.70	68.30	80.60
Minimum	22.00	14.00	18.00	15.00		21.80	26.00	27.30	28.90
Standard of Deviation	29.25	21.11	22.72	24.26		4.16	5.30	5.11	5.68
Coefficient of Variation (%)	34.28	36.13	36.72	36.54		12.78	12.99	11.71	12.97

**Table 4:** Dry period length of FH Cows in 1^st^, 2^nd^, 3^rd^, and 4^th^ lactation.

Dry Period Length	Lactation 1	Lactation 2	Lactation 3	Lactation 4
Average (days)	51.37	65.10	65.00	65.78
Maximum (days)	97.00	133.00	131.00	127.00
Minimum (days)	24.00	25.00	35.00	28.00
Standard of Deviation	9.33	22.69	20.49	21.60
Coefficient of Variation (%)	18.17	34.85	31.52	32.84

The values of genetic variance, environmental variance, and heritability are presented in Table 5, and the breeding values of sires are presented in Table 6.

**Table 5:** Inferred genetic variation, environmental variation, and heritability.

Model	Genetic Variation	Environmental Variation	$h^{2}$
Corrected milk yield	36895.8	43977.0	0.03

**Table 6:** Rankings of sires breeding value.

ID Sires	Breeding Value	Rankings
O.S.Elmer-XA	595.91	1
M.Z.Merlin.-XA	264.16	2
L.Muscadet-XA	252.38	3
C.Toyjet	247,12	4
S.GypsyB	239,01	5
WestCoastPldge	214,82	6
M.Z.Merlin-ET	188,14	7
Brasilia	178,56	8
L.JetBowser-XA	166,43	9
MRMUDD-XA	162,06	10

The production performance of FH dairy cattle is included in the good category, supported by 1st lactation data, which has an average milk production of 8,029.28 ± 1,112 kg; length of lactation 321.26 ± 38.48 days; peak production on day 85.36 ± 29.25 with milk production of 32.55 ± 4.16 kg; and the dry length period was 51.37 ± 9.33 days. Lactation 2 milk production 7,761.66 ± 1,145 kg; length of lactation 323.66 ± 43.06 days; peak production on day 58.43 ± 21.11 with milk production 40.79 ± 5.30 kg; and dry length period was 65.10 ± 22.69 days. Lactation 3 milk production 7,788.92 ± 1,148 kg; length of lactation 326.64 ± 46.74 days; peak production on day 61.88 ± 22.72 with milk production 43.62 ± 5.11 kg; and the dry length period was 65.00 ± 20.49 days. Lactation 4 milk production was 7,484.18 ± 1,133 kg, lactation length was 323.04 ± 42.23 days, peak production was on day 66.39 ± 24.26, milk production was 43.82 ± 5.68, and the dry length period was 65.78 ± 21.60 days. The heritability value of FH cow milk production namely 0.03 ± 0.02, is included in the low category. Differences in heritability values can be caused by differences in the type of data and statistical analysis used [10]. Low heritability estimates for the studied trait indicated that the differences in the performance traits of Holstein cows were mainly due to different nutritional, climatic conditions, and management practices prevalent over different times [11]. Ranking of 10 sire's that have the potential to increase the genetic potential of their offspring based on their estimated breeding value, namely 595.91 kg (O.S. Elmer-XA), 264.16 kg (M.Z. Merlin-XA), 252.38 kg (L. Muscadet-XA), 247.12 kg (C.Toyjet), 239.01 kg (S. Gypsy B), 214.82 kg (WestCoastPldge), 188.14 kg (M.Z. Merlin-ET), 178.56 kg (Brasilia), 166.43 kg (L.JetBowser-XA), and 162.06 kg (MRMUDD-XA).

## 4 Experimental Design, Materials And Methods

### 4.1 Materials Research

The object of the study was the complete record of milk production, lactation length, peak production, and dry period length of FH cows in lactation 1 to 4. Data were collected from the Ultra Farm company in South Bandung from 2017 up to 2021 based on the census method. Data were gathered from 675 cows in the first lactation, 599 cows in the second lactation, 591 cows in the third lactation, and 314 cows in the fourth lactation. Data were, then, descriptively and quantitatively analyzed.

### 4.2 Research Location

The research was carried out in Ultra Pangalengan, West Java, Indonesia at an altitude ranging from 1,400–1,500 m asl. The environment at the research location has a temperature and humidity index of 61.67 that is the comfort zone of FH cows. FH dairy cows will be comfortable at a THI value below 72. If the THI value exceeds 72, then FH dairy cows will experience mild stress (72≤THI≤79), moderate stress (80≤THI≤89), or severe stress (90≤THI≤97).

### 4.3 Experimental Design

This research used a case study method at an Ultra Farm company in South Bandung. Data were collected from 2017 up to 2021 using the census method, namely: milk production, length of lactation, peak production, and dry period length of dairy cows from the first lactation to the fourth lactation. The research was conducted in June 2023.

### 4.4 Data Collection

**Fig. 1:**
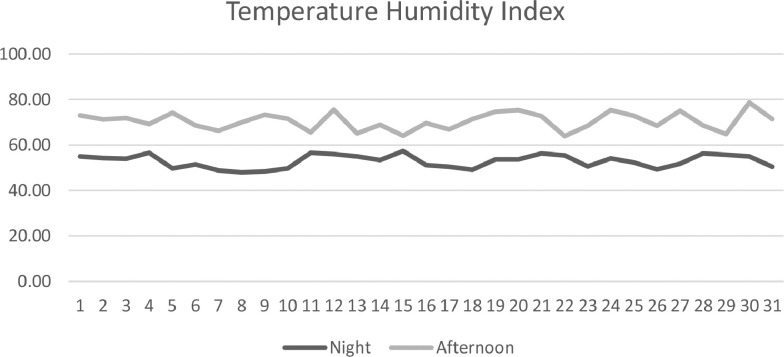
Temperature humidity index at the research location.

The data is tabulated and then sorted according to what is relevant, namely cow's number, dam, sire, date of birth, calving date, milk yield, length of lactation, peak production, and dry length period. Milk yield is the total amount of milk during lactation. Length of lactation, is the phase when the cow starts producing milk until it stops milking or the dry-stall phase. Peak production is the day and total milk when a lactation period reaches a high point in milk production. Dry period length, is the phase when cows stop producing milk until partus or calving (Fig. 1).

### 4.5 Data Analysis

The data was analyzed to estimate four statistical parameters, including variance, heritability, breeding value, and Temperature Humidity Index (THI). The statistical factors include dependent and independent variables. The dependent factor is milk production; whereas, the independent variables are length of lactation, peak production, and dry period length. Productivity performance in the form of four variables, then carried out the quantitative descriptive analysis, namely maximum value, minimum value, average, standard of deviation: $\sigma =\sqrt{\frac{\sum {\left(x_{i}-\mu \right)}^{2}}{N}}$ (σ= standard deviation of the population, x_i_ = Data value of i, μ = Average, N = Amount of data) and coefficient of variation: CV= $\frac{\sigma }{\mu }$ x 100% (μ =population average; σ=standard of deviation). Milk yield is corrected using a correction factor. The results of these calculations are used to measure heritability and breeding values. Milk production is standardized using United States Department of Agriculture (USDA) correction factors which have been modified according to Indrijani [12] as follows:

• The regression equation for a lactation length of less than 305 days becomes a lactation length of 305 days, for dairy cows aged less than or equal to 36 months.

Hoerl Model: ŷ=$\left(280.97692\right)\left(1.001079^{x}\right)\left(x^{\left(-1,0442258\right)}\right)$

Note: x = lactation length (days); ŷ = correction factors

• The regression equation for a lactation length of less than 305 days becomes a lactation length of 305 days, for dairy cows more than 36 months old.

Hoerl Model: ŷ=$\left(257.85161\right)\left(1.0015769^{x}\right)\left(x^{\left(-1,0569318\right)}\right)$

Note: x = lactation length (days); ŷ = correction factors

• The regression equation for a lactation length of more than 305 days becomes a lactation length of 305 days.

Hoerl Model: ŷ=$\left(0.00835972\right)\left(0.99381142^{x}\right)\left(x^{\left(1,1678976\right)}\right)$

Note: x = lactation length (days); ŷ = correction factors

• Regression equation to adjust cow age towards equivalent adult age.

4th Degree Polynomial Model:
  $$\begin{array}{ccc} \hat{y} & = & \left(1.8181749\right)+\left(-0.02794495\right)x+\left(0.000337177\right)x^{2}+\left(0.0000017241288\right)x^{3}\\ & & +\left(0.000000003373\right)x^{4} \end{array}$$

Note: x = age of cow (months); ŷ = correction factors

• The regression equation for milking frequency is twice the age of 2-3 years.
  $$\hat{y}=\left(0.991287356321839\right)+\left(-0.000539488320355951\right)x$$

Note: x = lactation length (days); ŷ = correction factors

• The regression equation for milking frequency is twice the age of 3-4 years.
  $$\hat{y}=\left(0.993724137931034\right)+\left(-0.000475639599555061\right)x$$

Note: x = lactation length (days); ŷ = correction factors

• The regression equation for milking frequency is twice age >4 years.
  $$\hat{y}=\left(0.994022988505747\right)+\left(-0.0004238042269188\right)x$$

Note: x = lactation length (days); ŷ = correction factors

Heritability was estimated using Variance Component Estimation (VCE) 6 software, and Breeding Value was estimated using Best Linear Unbiased Prediction (BLUP) using Parameter and Estimation (PEST) software. The formula used in this method is as follows:

y=Xb+Zu+e

Note: y = Observation vector (305 days cumulative production); X = Design matrix for fixed effects (lactation); Z = Design matrix for random effects; b = Vector for fixed effects; u = Vector for random effects; e = Vector of residues

The temperature and humidity index for dairy cows are calculated based on the research formula of Nascimento, et al., (2019):

THI = [3,43 + (1,058 x T) – (0,293 x RH)] + [(0,0164 x T x RH) + 35,7].

Note: THI: Temperature Humidity Index; Temperature (°C); Relative Humidity (%)

## Limitations

Not applicable.

## Ethics Statement

Our study focuses on population genetics utilizing statistical methodology. No animals were injured as a result of the study's findings. Industrial farms on which the research provides optimal living conditions for all animals.

## CRediT authorship contribution statement

**Prafangasti Sarah Ginantika:** Conceptualization, Resources, Methodology, Writing – review & editing. **Didin Supriat Tasripin:** . **Heni Indrijani:** Conceptualization, Supervision, Validation, Methodology. **Dedi Ruswandi:** Validation, Funding acquisition, Methodology, Writing – review & editing.

## Data Availability

Dataset for performance of superior dairy cattle sires (Original data) (Mendeley Data).
